# Synthesis of Copper Nitride Layers by the Pulsed Magnetron Sputtering Method Carried out under Various Operating Conditions

**DOI:** 10.3390/ma14102694

**Published:** 2021-05-20

**Authors:** Magdalena Wilczopolska, Katarzyna Nowakowska-Langier, Sebastian Okrasa, Lukasz Skowronski, Roman Minikayev, Grzegorz W. Strzelecki, Rafal Chodun, Krzysztof Zdunek

**Affiliations:** 1 National Centre for Nuclear Research, Material Physics Department, Plasma/Ion Beam Technology Division, 05-400 Otwock, Poland; sebastian.okrasa@ncbj.gov.pl (S.O.); grzegorz.strzelecki@ncbj.gov.pl (G.W.S.); 2 Institute of Mathematics and Physics, UTP University of Science and Technology in Bydgoszcz, 85-796 Bydgoszcz, Poland; lski@utp.edu.pl; 3 Laboratory of X-ray and Electron Microscopy Research, Institute of Physics PAS, Polish Academy of Sciences, 02-668 Warsaw, Poland; minik@ifpan.edu.pl; 4 Division of Surface Engineering, Faculty of Materials Science and Engineering, Warsaw University of Technology, 00-661 Warsaw, Poland; rafal.chodun@inmat.pw.edu.pl (R.C.); krzysztof.zdunek@pw.edu.pl (K.Z.)

**Keywords:** copper nitride, pulsed magnetron sputtering, plasma surface engineering method, Raman spectroscopy, optical properties

## Abstract

Copper nitride shows various properties that depend on the structure of the material and is influenced by the change in technical parameters. In the present work, Cu–N layers were synthesized using the pulsed magnetron sputtering method. The synthesis was performed under different operating conditions: direct current (DC) or alternating current (AC) power supply, and various atmospheres: pure Ar and a mixture of Ar + N_2_. The structural properties of the deposited layers were characterized by X-ray diffraction measurements, and Raman spectroscopy and scanning electron microscopy have been performed. Optical properties were also evaluated. The obtained layers showed tightly packed columnar grain features. The kinetics of the layer growth in the AC mode was lower than that observed in the DC mode, and the layers were thinner and more fine-grained. The copper nitride layers were characterized by the one-phase and two-phase polycrystalline structure of the Cu_3_N phase with the preferred growth orientation (100). The lattice constant oscillates between 3.808 and 3.815 Å for one-phase and has a value of 3.828 Å for a two-phase structure. Phase composition results were correlated with Raman spectroscopy measurements. Raman spectra exhibited a broad, diffused, and intense signal of Cu_3_N phase, with Raman shift located at 628–635 cm^−1^. Studies on optical properties showed that the energy gap ranged from 2.17 to 2.47 eV. The results showed that controlling technical parameters gives a possibility to optimize the structure and phase composition of deposited layers. The reported changes were discussed and attributed to the properties of the material layers and technology method.

## 1. Introduction

Copper nitride (Cu_3_N) layers exhibit promising physical properties, can be synthesized economically, and are nontoxic and highly stable, but only under room temperature. Their lattice constant often changes according to the production techniques. Therefore, an interesting feature of this material is that depending on the stoichiometry (lattice constant parameter) of the obtained Cu_3_N compound, it may exhibit properties typical of an insulator [1] or a semiconductor [1,2,3,4], and even those properties that are characteristically shown by a conductive material [5].

Successful synthesis of Cu_3_N is associated with difficulties related primarily to the narrow range of thermodynamic stability of the Cu_3_N phase, which decomposes at a high temperature. Large discrepancies are observed in terms of the decomposition temperature. According to previous studies [6,7,8], the decomposition temperature ranges from 100 to 450 °C. The reported differences are most likely related to the conditions of synthesis that determine the final structure of the material. It is known that in Cu_3_N synthesized using glow discharge with a magnetic field, the degree of material defects most likely affects the thermodynamic stability of the material. The range of thermodynamic stability of this material corresponds to the characteristic temperature of the I zone of the structural models [9]; low temperature during the synthesis and limited surface diffusion leads to an unfavorable structure of the material (defects and columnar structure). Heat activation, which results in synthesis, is hindered by the thermodynamic stability of the material. The practical range of synthesis of such materials is very narrow and difficult to observe. On the basis of our previous studies [10,11,12], plasma surface engineering could be used to synthesize the metastable Cu_3_N material with reduced thermal activation. In the pulsed magnetron sputtering (PMS) method, the synthesis process is implemented in a pulsed manner, which enables it to be substituted for different forms of energy during synthesis. In addition, this method gives visibility to a greater degree of the parameters, such as electrical energy resulting from the participation of energetic species and electric field during the process of synthesis, which means a non-equilibrium plasma state.

The main objective of the present paper was focused on the technology of copper nitride layers synthesis, i.e., change of the technological parameters, such as working mode AC/DC of pulsed power supply and gas environment, to obtain structure and optimization of deposition processes in terms of the desired quality of the obtained material. Production of high-quality material with a single-phase Cu_3_N structure is important from the point of view of, firstly, the verification of the adopted hypothesis regarding the scope of possibilities of searching for other synthetic pathways, and secondly, it opens up new application perspectives by using the base material with a stabilized phase composition.

## 2. Materials and Methods

The material was synthesized using the PMS method. Cu_3_N layers were synthesized by the circular magnetron WMK-50 (IMT, Wroclaw University of Technology, Poland) (according to the Gencoa classification of balanced/unbalanced magnetrons where distance to the null point (Z_BZ_) is 28 mm, g = 1.27 [13,14,15]). A 50-mm diameter and a 6-mm thick copper target (Grade 1 purity) was used as a magnetron cathode. This method enabled the material to be synthesized on a cold surface. Moreover, there was no risk of thermal decomposition of the Cu–N layer.

**Figure 1:**
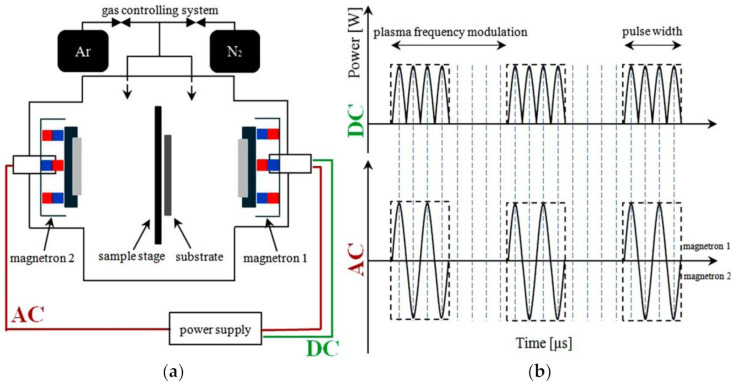
(**a**) Schematic diagram of the apparatus used in the experiment; (**b**) the scheme of current pulse time behavior in the AC and DC modes of pulsed power supply.

The magnetron cathode was powered by a 10 kW DORA PS pulsed power supply [16,17], operated in two modes: DC and AC [18,19]. This device controls the modulation of carrier frequency [20]. Figure 1 shows a schematic illustration of the experimental setup and operation of the power supply system. The schematic overview in Figure 1b shows the differences in the operation of the power supply system in AC and DC modes. The figure illustrates the plasma pack generation controlled by pulsed width modulation.

The DORA PS power supply could operate in both AC and DC modes. The AC mode is provided by a super-modulated sine wave with 50% duty cycle. In the DC mode, the negative half-periods of the signal are transferred to the positive side of the waveform. In the AC mode, the two magnetrons were working alternately (the magnetrons were mounted in the chamber in mirror configuration, but only one magnetron was used during the experiment to synthesize our samples). This implies that only the positive part of the AC waveform was involved in plasma generation. Thus, the plasma contribution in the bipolar process of film synthesis was half of that of the unipolar process. The ability to work in these two modes reduced the share in the synthesis of plasma, which implies that the life span of plasma was shortened by half, while retaining the other synthesis parameters at the same level.

Cu_3_N layers were deposited on non-heated Si (100) (Lukasiewicz Research Network—Institute of Electronic Materials Technology, Warsaw, Poland), and glass substrates (SIMAX, Blachownia, Poland) by the PMS method [16]. The substrates were ultrasonically cleaned (Codyson 0,6l 50W CD3800, Shenzhen Codyson Electrical Co., Guangdong, China) in acetone (Linegal Chemicals, Warsaw, Poland) and mounted on a stage located at a distance of d_S-T_ = 10 cm from the magnetron cathode. The substrate surface was oriented perpendicularly to the *Z*-axis of the magnetron cathode. During insertion of the substrate, the chamber is opened and the target is exposed to impurities that can get onto its surface. In order to remove contaminants from the target pre-sputtering processes are carried out. A cathodic material was pre-sputtered in a 1.2 Pa argon atmosphere for 10 min before every layer deposition process.

Cu_3_N layers were fabricated in four variants: DC and AC power supply modes and various gas atmospheres. The copper cathode was sputtered with 80 W power in an atmosphere of pure nitrogen (N_2_) and its mixture with argon (N_2_ + Ar). For AC processes, the sputtering time was extended to twice the normal time because the generation time of plasma was reduced by half (Figure 1). The time of sputtering was set to 60 and 120 min for DC and AC modes, respectively, to compare the deposited layers (Table 1).

The surface of the deposited layers was characterized by scanning electron microscopy (SEM, Zeiss, Jena, Germany). The chemical composition (at.%) of the Cu–N layers was analyzed by an energy-dispersive X-ray (EDS) analyzer (SEM, Zeiss, Jena, Germany), 20 kV, working distance 10 cm). The phase composition of the Cu–N layers was analyzed by an X-ray diffraction technique, with help of an laboratory diffractometer X’Pert Pro Alpha1 MPD (XRD, Malvern-Panalitical, Almelo, Netherlands) equipped with Cu-Kα X-ray tube. The layers were measured in the 2θ scan mode. To confirm phase composition, oscillation spectroscopy was performed using a Raman Jasco NRS 5100 spectrometer (Jasco, Tokyo, Tokyo Metropolitan Prefecture, Japan) operating in backscatter geometry and using 532 nm argon ion laser.

Optical studies of the Cu–N layers on silicon and glass substrates were performed by spectroscopic ellipsometry (a rotating analyzer device: V-VASE device, J.A. Woollam Co., Inc., Lincoln, NE, USA) and spectrophotometry (a Cary 5000 unit, Agilent, Santa Clara, CA, USA). The ellipsometric parameters Ψ and Δ azimuths were recorded in the photon energy spectral range from 0.62 eV (2000 nm) to 5 eV (248 nm), with a resolution of 0.02 eV for three angles of incidence (65°, 70°, and 75°). Transmittance spectra (*T*) were acquired for a near-normal incidence of light for the wavelength region from 248 nm to 2000 nm (the resolution for this measurement was 2 nm). These measurements allowed us to obtain refractive index (*n*) and extinction coefficient (*k*). The optical band gap energy was obtained by extrapolating the absorption edge line with abscissa to the low energy.

## 3. Results

### 3.1. Structure and Phase Composition of Copper Nitride

**Figure 2:**
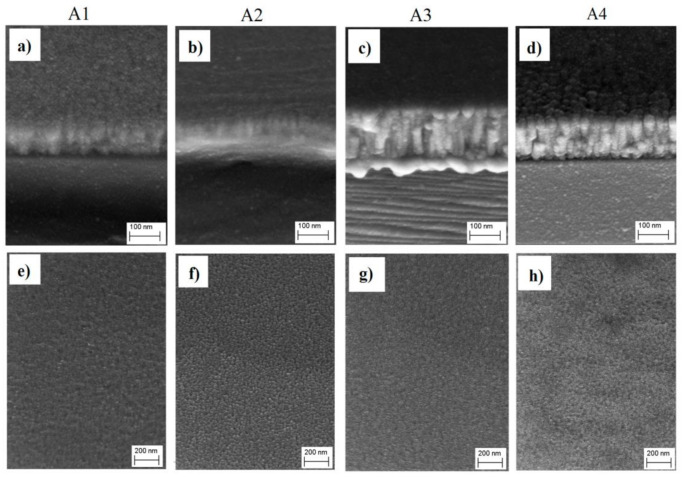
SEM images of Cu–N layers synthesized with various process parameters (**a**–**d**) cross-section; (**e**–**h**) surface morphology.

Figure 2 shows cross-section and surface morphology images of the Cu–N layers prepared with various process parameters. In terms of morphology, the obtained layers showed tightly packed columnar grains features and sharp grain boundaries; this characteristic is commonly observed in layers obtained by magnetron sputtering and agrees well with the zone transition (T) microstructure of Thornton’s model [9]. All the layers showed clean and smooth surface morphology. The working mode (AC or DC) had more influence on the morphology and structure of the layer than the plasma working gases. The kinetics of the layer growth in the AC mode, despite the use of a longer time (to compensate for the loss of plasma generation to the second magnetron), were lower than that observed in the DC mode. The AC mode caused the layers to be thinner and more fine-grained than those obtained in the DC mode [21].

**Figure 3:**
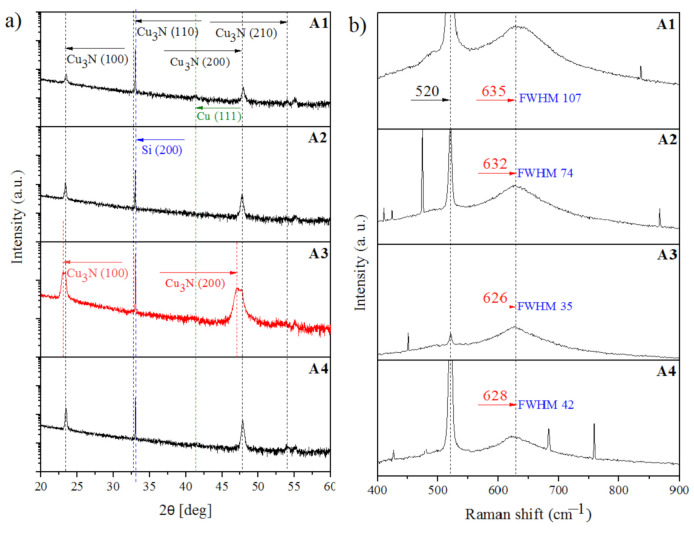
(**a**) The X-ray diffraction patterns and (**b**) Raman spectra of the Cu–N layers.

Figure 3a shows the X-ray diffraction patterns of the Cu–N layers. The obtained Cu–N layers had an anti-ReO_3_ polycrystalline structure with a privileged orientation, with the preferred growth orientation along the direction (100) (Figure 3). Diffraction studies enabled the calculation of the lattice constant. The three layers that met the requirements of a single-phase structure showed results similar to the literature data on the Cu_3_N phase [17]. The lattice parameter (a_0_) of the synthesized layers was calculated from the X-ray peak position of the lattice plane reflex (hkl), and it was found to oscillate between 3.808 and 3.815 Å, according to the values from the literature for stoichiometric single crystal Cu_3_N = 3.815 Å [2,22,23]. Bragg peaks corresponded to the Cu_3_N crystalline structure. In addition, a Cu(111) reflection of the Cu capping layer was observed. The mean grain size of the layers was estimated by the Scherrer formula, and the results are presented in Table 2. These layers are stoichiometric, single phase, with similar grain size values of 30–35 nm. However, a two-phase structure was obtained for one of the layers. The peaks recorded on the diffractogram corresponded to two polycrystalline phases that crystallized in the structure of the ReO_3_-copper nitride type, but with different stoichiometries. One of them corresponded to the phase of stoichiometric copper nitride with a small crystallite size of 15 nm. For the second phase, the peaks shifted to the lower values of the angle 2θ, which corresponded to Cu_3_N supersaturated with copper (Cu) (Figure 3a). This phase had a larger lattice constant of 3.828 Å and a bigger size of grains. The layer was probably stoichiometric with cubic Cu_3_N and regions of Cu_3_N saturated with copper; this is because when more Cu was interstitially doped, Cu_3_N was retained as a cubic structure with a larger lattice constant. In terms of the technical process, this is most likely due to the more intensive spraying of the copper target, along with energy conditions prevailing on the surface. The above discussion demonstrates that XRD diffraction patterns of Cu_3_N show no dependence on the structure of synthesized layers on various PMS process parameters.

Phase composition obtained through the X-ray diffraction method was confirmed by the results obtained from the Raman spectroscopy method. Figure 3b shows the obtained spectra recorded for the Cu–N layers. The crystal structure of Cu_3_N belongs to the space group, Pm-3m, wherein the unit cell contains one formula unit. The space group analysis predicts 12 phonon modes at Γ point, out of which nine optic modes with symmetry representation are as follows: Γ = 2F_1u_ + F_2u_. The F1u modes are infrared (IR) active and F2u modes are optically inactive (silent mode). Hence, no first-order Raman signal is expected for a perfect cubic Cu_3_N. However, the experimental results available for Raman spectra of this material are quite debatable; they show the presence of Raman peak at 634 cm^−l^ for Cu_3_N thin films [12,24,25,26,27]. Though the theoretical calculation inhibits the presence of Raman active modes for a perfect cubic Cu_3_N, the possibility of modes arising because of the selection rule break down due to non-stoichiometry cannot be ruled out. These modes arise because of the selection rule breakdown owing to the highly non-stoichiometric nature (due to the presence of defects and disorders at Cu and N sites) and multi-phonon combinations. Consideration of the results of Raman shift at 634 cm^−1^ for Cu_3_N films and XRD complement each other and confirm our predictions. However, a more detailed theoretical analysis dedicated to the exact assignment of Raman-active peak would be required for a proper interpretation.

Considering the above reasons, perfect copper nitride is very difficult to synthesize. However, based on literature data and other research methods, such as XRD and the examination of optical properties, we are confident that obtained layers are copper nitride. Raman shift around 635 cm^−1^ corresponds to copper nitride which, however, has no perfect cubic Cu_3_N lattice with the symmetry group (Pm-3m). The Raman shift located at 520 cm^−1^ originated from the silicon substrate on which the layer was synthesized. Raman spectra exhibited a broad, diffused, and intense fluorescence signal of Cu_3_N. The position of peaks and FWHM (full width at half maximum) and shape were optimized by the Voight function in OriginLab (OriginPro 7.5, OriginLab Corporation, Northampton, MA, USA). All results were fitted and are listed in Table 2. The results of Raman spectroscopy also showed a greater effect of the working mode (AC or DC) on the structure than the yield-generating gases. Considering the change in the working mode, the Raman shift of the layers deposited by the AC mode was similar to that found in the literature data. However, the spectrum obtained from the DC mode was not significantly different. This spectrum was shifted to lower wavenumbers. The FWHM values were dependent on the working mode and showed whether the layer was crystalline or amorphous. The Raman spectra of crystalline and amorphous solids of the same chemical composition can be significantly different, primarily because of the presence or absence of spatial order and long-range translational symmetry, respectively. Amorphous solids can be thought of as a collection of formula units of the same chemical composition, but with varying bond angles and lengths depending upon chemical bond interactions with their nearest neighbors. There is no order to their arrangement in space. Consequently, one does not observe the narrow bands. One can clearly observe that the FWHM results of Cu–N layers obtained by pulsed magnetron sputtering show wide bands, typical for amorphous layers. However, the value of FWHM of the layers obtained by the DC mode is significantly lower than from AC mode. It is known that widths depend upon the degree of chemical interaction between the molecules. Therefore, it can be assumed that working mode may impact the structure of copper nitride layers. Differences in Raman shifts in the results obtained by various PMS process parameters could be a reason for subtle structural changes. The Raman shift of the A1 sample at 635 cm^−1^ obtained under the AC mode and Ar + N_2_ gas atmosphere was consistent with the Cu_3_N peaks reported in the literature. The Raman spectra of the A3 sample did not clearly show any differences in the obtained results for the two-phase layer. This could be explained by the fact that the Raman laser spot diameter was 0.6 μm, and it was difficult to find areas with two phases when performing a measurement.

### 3.2. Optical Properties of the Cu_3_N Layers

**Figure 4:**
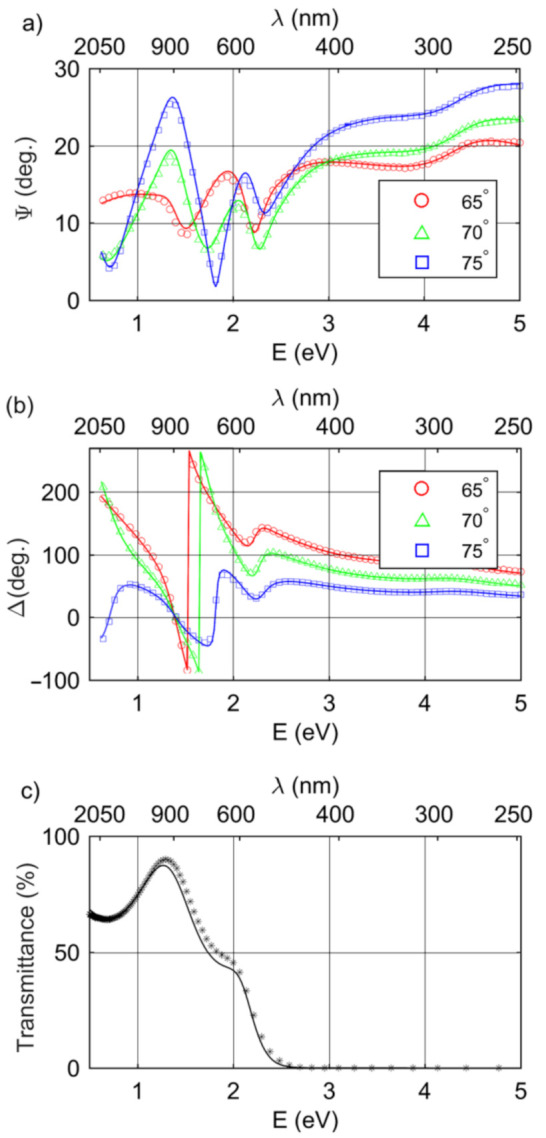
Elllipsometric azimuths (**a**) Ψ and (**b**) Δ and (**c**) transmittance spectra for A4 sample. Experimental data (circles, squares, triangles, and stars) are plotted every third (Ψ and Δ) or tenth (T) collected point. Solid lines represent the spectra calculated from the optical model of sample.

Optical constants of the examined Cu_3_N coatings and the thicknesses of the Cu–N and rough layers were determined using a four-medium optical model of the sample (ambient/rough layer/Cu–N layer/glass substrate) by combining both ellipsometric (Ψ and Δ) and spectrophotometric (*T*) measurements. Optical constants of Cu–N were parameterized using sum Gaussian-(${\tilde{\varepsilon }}_{G}$) and/or Lorentzian-type (${\tilde{\varepsilon }}_{L}$) oscillators [28,29], as well as PSEMI-M1 (${\tilde{\varepsilon }}_{PSEMI}$) shape of absorption line near the optical band gap [28]. Additionally, a Drude (${\tilde{\varepsilon }}_{D}$) term was considered. Generally, the complex dielectric function ($\tilde{\varepsilon }$) of the produced Cu–N layers can be written as follows:

(1) $$\tilde{\varepsilon }={\left(n+ik\right)}^{2}=\varepsilon_{\infty }+{\tilde{\varepsilon }}_{D}\left(\hbar \omega_{p},\hbar \Gamma \right)+{\tilde{\varepsilon }}_{PSEMI}\left(A,E,B,WL,WR,AL,AR\right)+\underset{j}{\sum }{\tilde{\varepsilon }}_{L,G}\left(A_{j},E_{j},Br_{j}\right)$$

where *n* is the real part of the complex refractive index, *k* is the extinction coefficient, $\varepsilon_{\infty }$ is a high-frequency dielectric constant, and $\hbar \omega_{p}\;$ and ℏΓ  are the plasma energy and free-carrier damping, respectively. In Equation (1), *A*/*A*_j_ is the amplitude, *E*/*E*_j_ is the energy of the absorption line, and *Br*_j_ is the broadening of the oscillator. Parameters *B*, *WL*, *WR*, *AL*, and *AR* are associated with the broadening and shape of the PSEMI-M1 absorption line. The analytical formulas related to the particular oscillators are described in [28,29]. Optical constants of the substrate were taken from the database of optical constants [28]. Optical properties of the rough layer were described as a Bruggeman Effective Medium Approximation [28,29], with fractions of void and Cu–N equal to 50%. The parameters of particular absorption lines and thicknesses of the rough (*d*_r_) and Cu–N layers (*d*_Cu–N_) were varied to minimize the reduced mean squared error ($\chi^{2}$) defined as [28,29]:

(2) $$\chi^{2}=\frac{1}{N_{\Psi \Delta }-P}\underset{j}{\sum }\left[{\left(\frac{\Psi_{j}^{\mathrm{mod}}-\Psi_{j}^{\exp}}{\sigma_{j}^{\Psi }}\right)}^{2}+{\left(\frac{\Delta_{j}^{\mathrm{mod}}-\Delta_{j}^{\exp}}{\sigma_{j}^{\Delta }}\right)}^{2}\right]+\frac{1}{N_{T}-P}\underset{j}{\sum }\left[{\left(\frac{T_{j}^{\mathrm{mod}}-T_{j}^{\exp}}{w_{T}\sigma_{j}^{T}}\right)}^{2}\right]$$

where $N_{\Psi \Delta ,T}$ and *P* are the total number of data points and the number of fitted model parameters, respectively. The variables with superscripts exp and mod correspond to the experimental and calculated data, respectively. In Equation (2), $w_{T}\;$ is weighting of *T* values, while variables $\sigma_{j}^{\Psi }$, $\sigma_{j}^{\Delta }$, and $\sigma_{j}^{T}$ are standard deviations of Ψ, Δ, and *T* data, respectively. The procedure of fitting was performed using the WASE32 software (version 3.774, J.A. Woollam Co., Inc., Lincoln, NE, USA) [28]. An example of the fit (for sample A4) is presented in Figure 4.

Thicknesses of the Cu–N layers estimated during the analysis of spectroscopic ellipsometry combined with transmittance data summarized in Table 3 were in the range of approximately 80 nm (A1) to approximately 220 nm (A3). The thicknesses of the rough layers (2.9–9.7 nm; see Table 3) did not exceed 10% of the thickness of the Cu–N layers.

**Figure 5:**
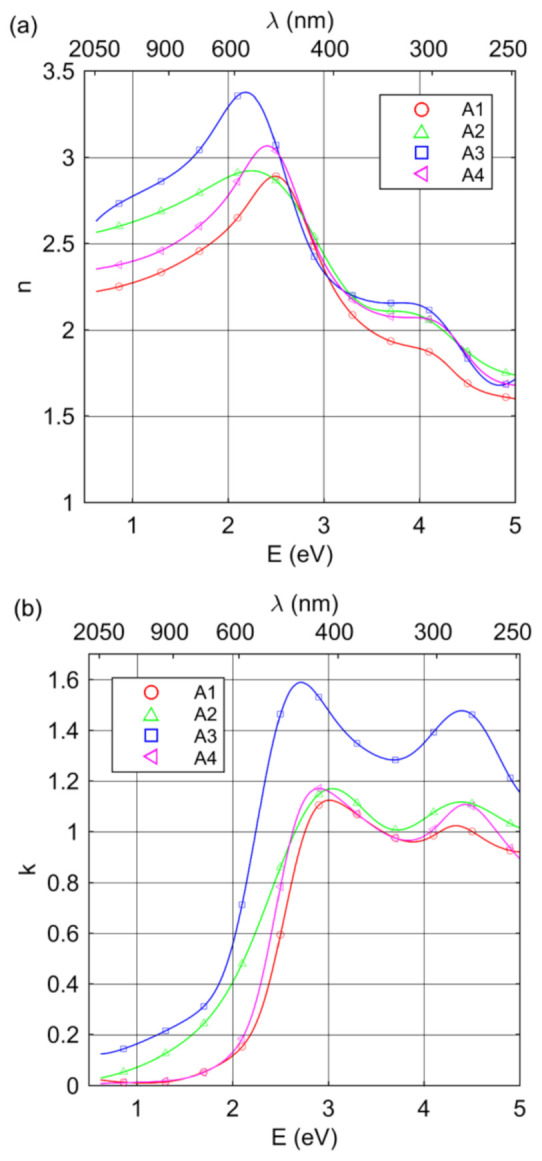
(**a**) Real part of the complex refractive index and (**b**) extinction coefficient of the Cu–N layers.

Figure 5 shows the real part of the complex refractive index and extinction coefficient of the synthesized Cu–N layers. In general, the shape of *k* with two maximums at 2.5–3.0 eV and 4.3 eV is typical for Cu–N layers and was reported earlier. In the IR spectral range, the extinction coefficient values were relatively small, especially for A1 and A4 samples. This behavior of *k* for long wavelengths was directly associated with the phase composition of Cu–N layers, particularly with the non-stoichiometry of Cu_3_N, or existence/coexistence of the pure Cu phase and/or the Cu_3_N phase supersaturated with Cu. The Drude term describes the interaction of electromagnetic radiation with free carriers. During the analysis of optical spectra, the Drude term was found only for samples produced under Ar + N_2_ mixture. For specimens deposited under pure N_2_ atmosphere, the plasma energy reached a value of zero during the fit procedure. The increase in *k*, with the increase in the wavelength (for λ > 1200 nm) for sample A1, and the relatively high value of *k* for sample A3, were associated with absorption caused by free carriers. The values of plasma energy were relatively small (0.27 and 0.58 eV for samples A1 and A3, respectively). The free carrier damping was 0.79 and 0.10 eV for samples A1 and A3, respectively; however, the uncertainty of the first value was high (approximately 50% of ℏΓ). On the basis of the estimated $\hbar \omega_{p}$ and ℏΓ values, the optical resistivity (ρopt=Γε0ωp; $\varepsilon_{0}$–permittivity of vacuum) and mean relaxation time of free carriers ($\tau =\Gamma^{-1}$) were calculated (Table 3). The values of $\rho_{opt}\;$ = 80 kΩ cm and τ = 0.8 fs for sample A1 were determined with high uncertainty (approximately 50%). The mean relaxation time of free carriers and optical resistivity for sample A3 were 6.4 fs and 2.3 kΩ cm, respectively. The conductivity of the Cu–N layers was associated with the insertion of Cu atoms into the body center of the Cu_3_N structure. This result agrees well with XRD measurement results, where, apart from the Cu_3_N structure, the Cu_3_N phase supersaturated with Cu was found.

**Figure 6:**
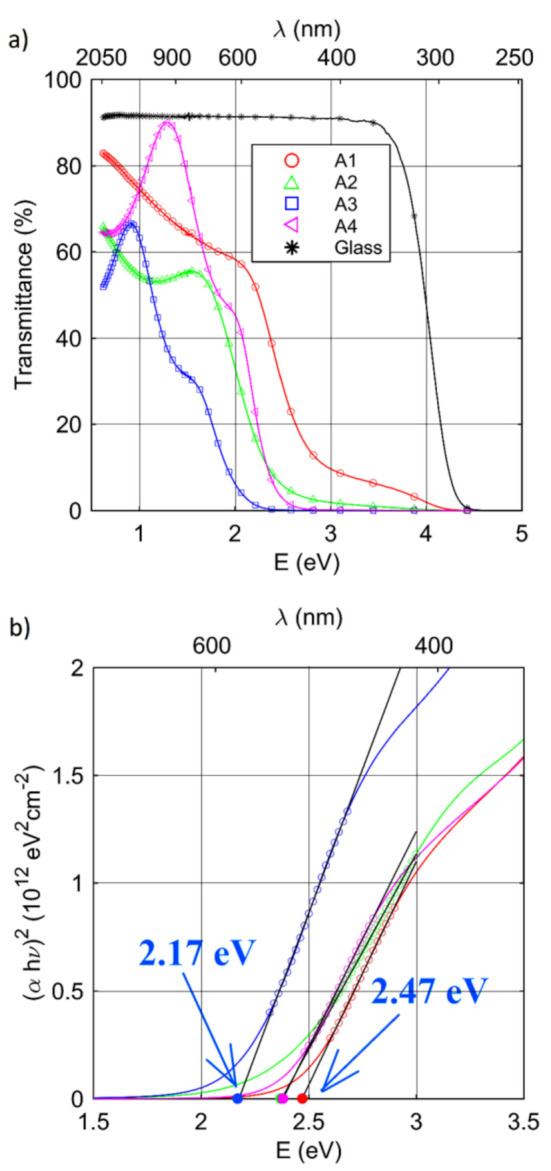
(**a**) Transmittance spectra recorded for the Cu–N layers. The data for glass are added for comparison; (**b**) The Tauc plot for the Cu–N layers.

Transmittance (*T*) spectra of the prepared Cu–N layers are presented in Figure 6a. For the glass substrate, the transmittance was 90% in the non-absorbing spectral range and decreased to zero in the spectral range from 3.5 to 4.5 eV. For the synthesized Cu–N layers, the *T* values were drastically decreased from approximately 1.5 eV (A3) to approximately 2.5 eV (A1). The shape of the transmittance spectra for lower photon energies resulted from interference of light in the thin Cu–N coating weakened by relatively low (but existing) absorption.

The Tauc method was used to estimate the band-gap energy ($E_{g}$) of the Cu–N layers [18]. According to the following relationship [18]:

(3) $$\left(\alpha h\nu \right)=B{\left(h\nu -E_{g}\right)}^{m}$$

the value of $E_{g}$ can be extracted by plotting ${\left(\alpha h\nu \right)}^{1/m}$ as a function of hν. In Equation (3), α is the absorption coefficient (α=4πk/λ), hν is photon energy, *B* is the band tailing parameter, and *m* is the parameter associated with the type of transition (*m* = 1/2 for a direct allowed transition, *m* = 3/2 for a direct forbidden transition, *m* = 2 for an indirect allowed transition, and *m* = 3 for an indirect forbidden transition) [18]. Gordillo reported that the allowed direct optical transition is observed for Cu–N layers [4]; therefore, *m* = 1/2 was chosen for this analysis. Figure 6b shows the Tauc plot for the investigated samples. The determined optical band-gap energies are summarized in Table 3 and depicted in Figure 6. *E*_g_ was in the range of 2.17 eV (A3) to 2.47 eV (A1). For samples deposited under pure N_2_ plasma, the value of *E*_g_ was practically the same (2.37–2.38 eV). The obtained band-gap energy values were higher than those reported earlier [2,4,21,30] where *E*_g_ ranged from 0.23 to 1.9 eV. However, a previous study [31] showed that higher energy maxima up to 2.46 eV is solely related to the large lattice mismatches and the strained Cu_3_N in immediate vicinity of the surface.

## 4. Conclusions

This work provides key findings regarding the morphology, phase composition, and properties of the Cu_3_N layers synthesized by the PMS method with various process parameters. The results of our studies showed that the change of the technological parameters, such as working mode AC/DC and gas environment, has an impact on the obtained structure of Cu–N layers. The X-ray diffraction study revealed a polycrystalline structure of Cu_3_N, with one or two phases with preferred growth along the direction (100). The calculated lattice constant was within the range of 3.808 to 3.817 Å and 3.838 Å for the one-phase and two-phase structures of the layers, respectively. The Raman spectroscopy measurements confirm phase composition results (Raman shift around 634 cm^−1^ corresponds to Cu_3_N phase) and provide useful information on the subtle structural changes in obtained layers. Studies on optical properties showed that the energy gap ranged from 2.17 to 2.47 eV.

## Figures and Tables

**Table 1:** PMS process parameters for synthesizing Cu–N layers.

Sample Name	Material	Working Mode	Gas Atmosphere	Pressure (Pa)	Time (min)	Power (W)	d_S-T_ (cm)
A1	Cu–N layers	AC	Ar + N_2_	2 (0.3 Ar)	120	80	10
A2	Cu–N layers	AC	N_2_	2	120	80	10
A3	Cu–N layers	DC	Ar + N_2_	2 (0.3 Ar)	60	80	10
A4	Cu–N layers	DC	N_2_	2	60	80	10

**Table 2:** Phase composition, N/Cu by EDS, lattice constant crystallite size, and Raman shift of the synthesized Cu–N layers according to various PMS process parameters.

Sample Name	Phase Composition	N/Cu by EDS (at.%)	Lattice Constant a_0_ (Å)	Crystallite Size D (nm)	Raman Shift (cm^−1^)	FWHM (cm^−1^)
A1	Cu_3_N	23.05/76.95	3.808 ± 0.00031	35	635 ± 2	107 ± 1
A2	Cu_3_N	24.89/75.11	3.815 ± 0.00021	33	632 ± 1	74 ± 2
A3	Cu_3_N	21.78/78.22	3.813 ± 0.00012	35	626 ± 2	35 ± 1
	Cu_3_N (Cu)		3.828 ± 0.00017	15		
A4	Cu_3_N	23.38/76.62	3.813 ± 0.00023	30	628 ± 1	42 ± 1

**Table 3:** Thicknesses of rough (*d*_r_) and Cu–N (*d*_Cu–N_) layers, Drude parameters (ℏω_p_, ℏΓ), mean relaxation time (τ), optical resistivity ($\rho_{opt})$.

Sample Name	A1	A2	A3	A4
*d*_r_ (nm)	2.9 ± 0.1	9.7 ± 0.1	4.8 ± 0.2	9.1 ± 0.1
*d*_Cu–N_ (nm)	79.6 ± 0.2	115.3 ± 0.2	222.1 ± 0.6	192.4 ± 0.2
$\hbar \omega_{p}$(eV)	0.27 ± 0.02	-	0.58 ± 0.05	-
ℏΓ(eV)	0.79 ± 0.39	-	0.10 ± 0.03	-
τ(fs)	0.8 ± 0.4	-	6.4 ± 1.5	-
$\rho_{opt}$(kΩcm)	80 ± 42	-	2.3 ± 0.6	-
*E*_g_ (eV)	2.47 ± 0.03	2.37 ± 0.03	2.17 ± 0.03	2.38 ± 0.02

## Data Availability

Data are contained within the article.
